# Supplementary material for: Influence of Ionizing Radiation on Spontaneously Formed Aggregates in Proteins or Enzymes Solutions
Source: Pharmaceutics. 2023 Apr 29;15(5):1367. doi: 10.3390/pharmaceutics15051367 (PMC10221975; doi:10.3390/pharmaceutics15051367)
Supplement: Supplementary file 1 [file pharmaceutics-15-01367-s001.zip › pharmaceutics-2295408-supplementary.pdf]

Supplementary Materials

Karolina Radomska, Marian Wolszczak *

* Correspondence: marian.wolszczak@p.lodz.pl

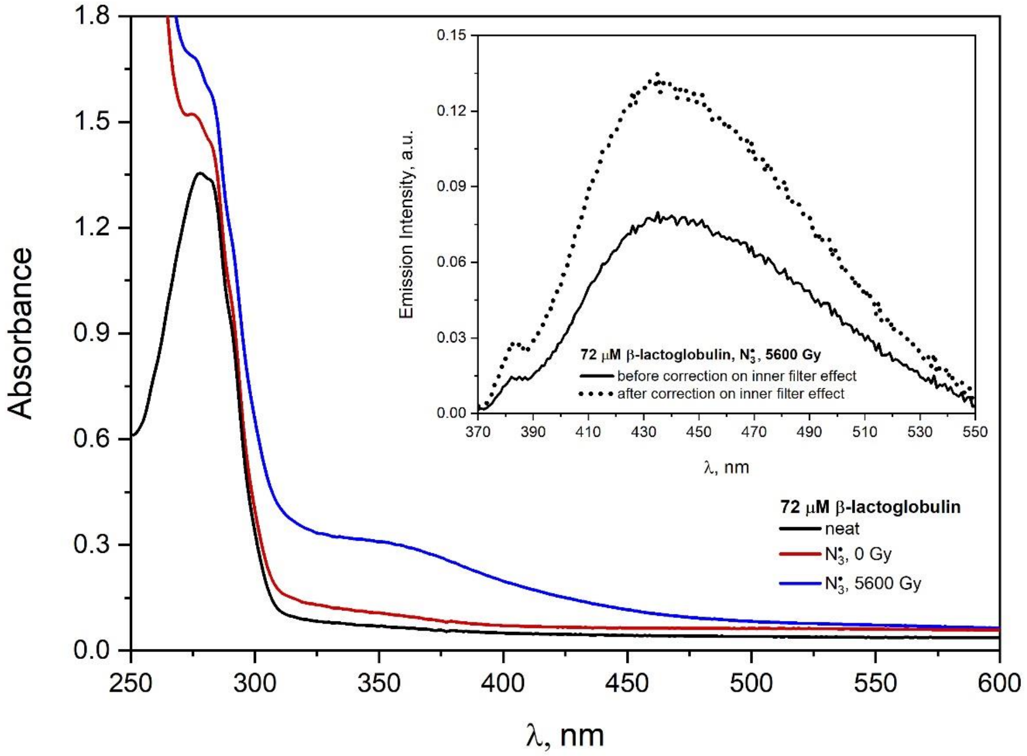

Figure S1. Absorption spectra of N_2O-saturated β-lactoglobulin solution (72 μM) containing NaN_3 (0.1 M) before and after irradiation with dose 5600 Gy. **Insert**. Emission spectrum of N_2O-saturated buffer β-lactoglobulin solution (72 μM) containing NaN_3 (0.1 M), obtained for irradiation dose 5600 Gy, before and after correction on inner filter effect.

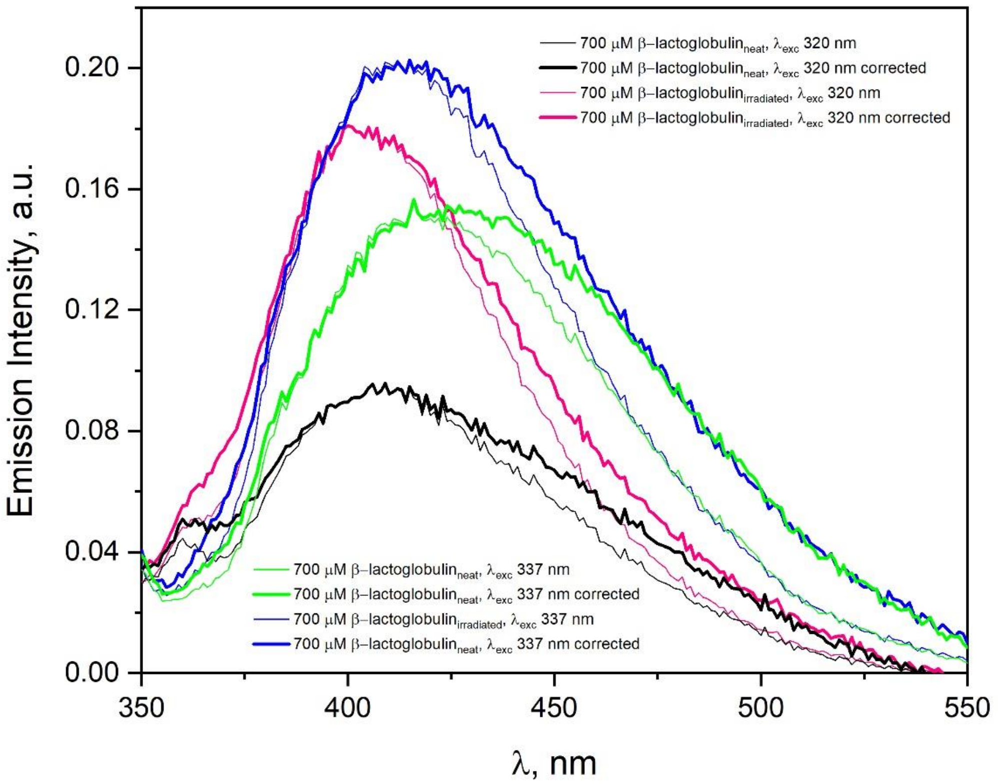

Figure S2. Emission spectra of neat β-lactoglobulin (700 μM) solution and N_2O-saturated buffer β-lactoglobulin solution (700 μM) containing NaN_3 (0.1 M), obtained for irradiation dose 1000 Gy (thin curves). The excitation wavelength was 320 or 337 nm. The thick curves represent the β-lactoglobulin emission spectra after correction for instrument response (mainly photomultiplier spectral response).

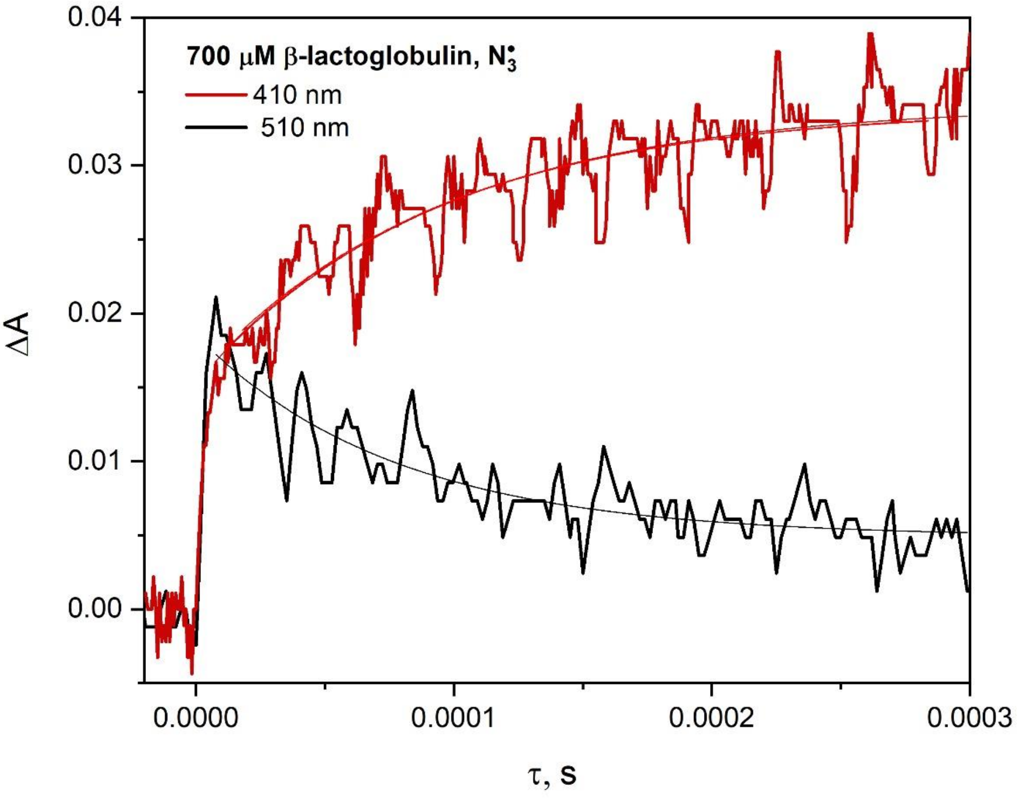

Figure S3. Time profiles of the absorbance recorded at 410 or 510 nm after irradiation with a dose of 200 Gy of the N_2O-saturated aqueous solution containing β-lactoglobulin (700 μM) and NaN_3 (0.1 M).

Table S1. The amount of Tyr and Trp residue in the structure of proteins and enzymes.

	**M**	**Trp**	**Tyr**
**HSA**	66.3 kDa	1	17
**BSA**	66.4 kDa	2	21
**OVA**	45.0 kDa	3	10
**papain**	23.4 kDa	5	19
**histone**	21.0 kDa	0	14
**β-lactoglobulin**	18.4 kDa	2	4
**lysozyme**	14.4 kDa	6	3
**insulin**	6.0 kDa	0	4
**VEALYL**	660 Da	0	1

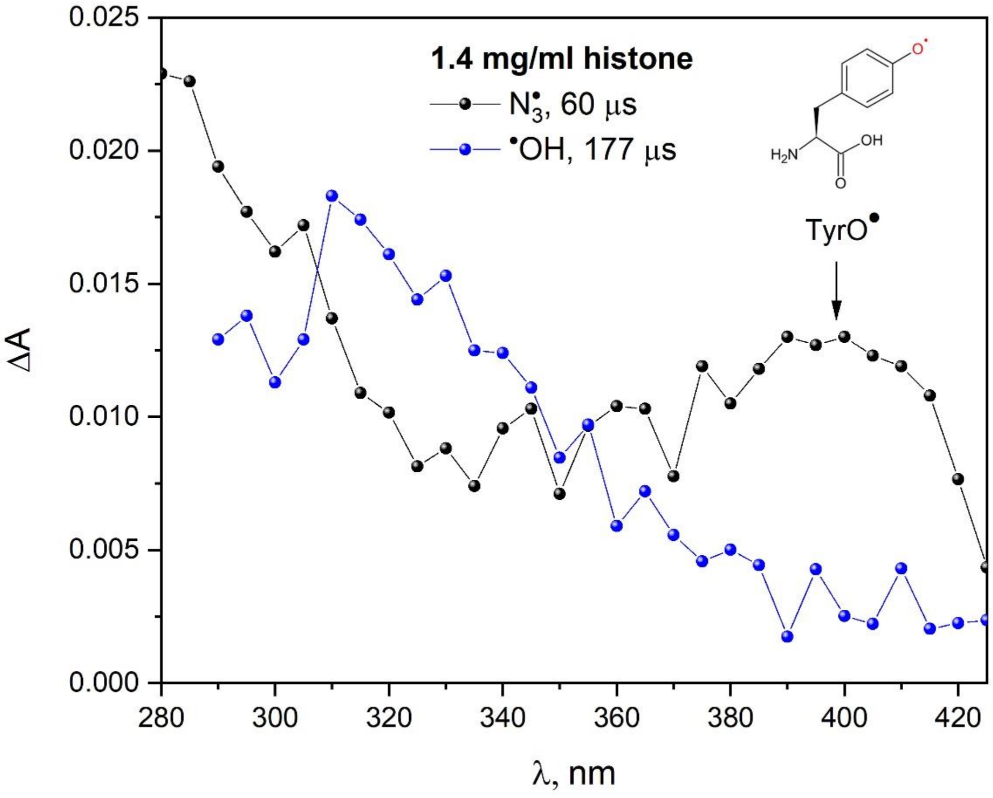

Figure S4. Transient absorption spectra of N_2O-saturated buffer solutions containing 1.4 mg/ml histone and 0.1 M NaN_3, obtained for an irradiation dose of 55 Gy.

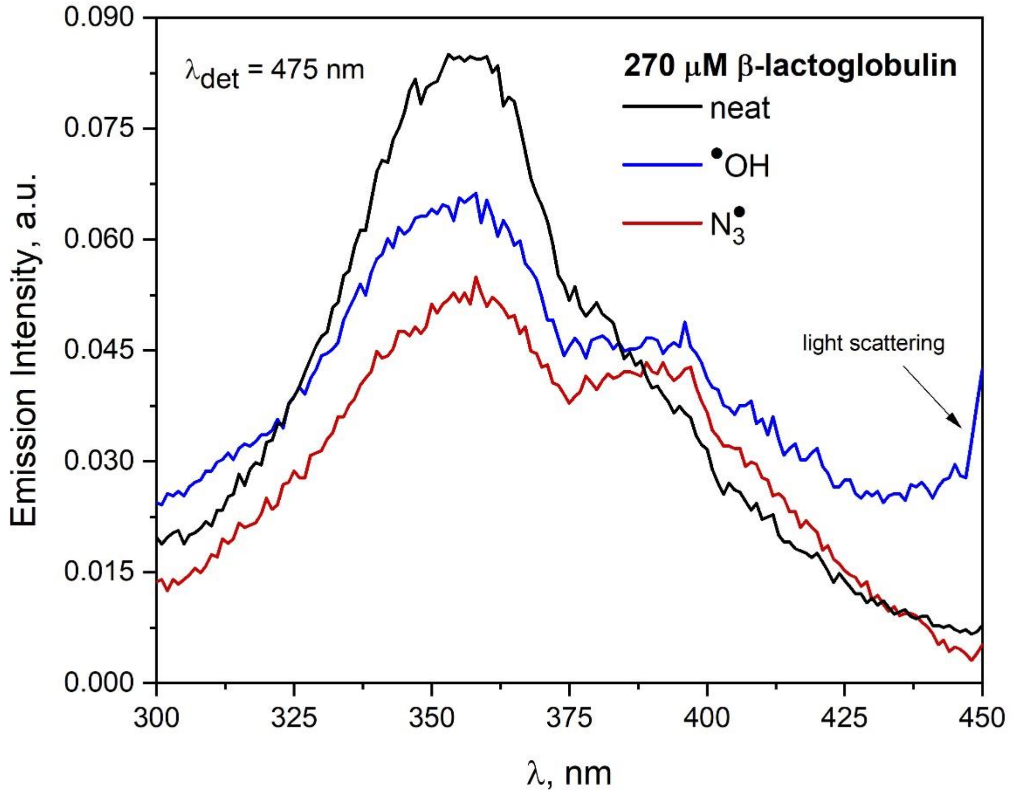

Figure S5. Emission excitation spectra of the N_2O-saturated β-lactoglobulin solution (270 μM), recorded before irradiation (black line). Emission excitation spectra of the N_2O-saturated β-lactoglobulin solution (270 μM), recorded after irradiation with a dose 24 kGy (blue line). Emission excitation spectra of the N_2O-saturated β-lactoglobulin solution (270 μM) containing NaN_3 (0.1 M), recorded after irradiation with a dose 24 kGy (red line).

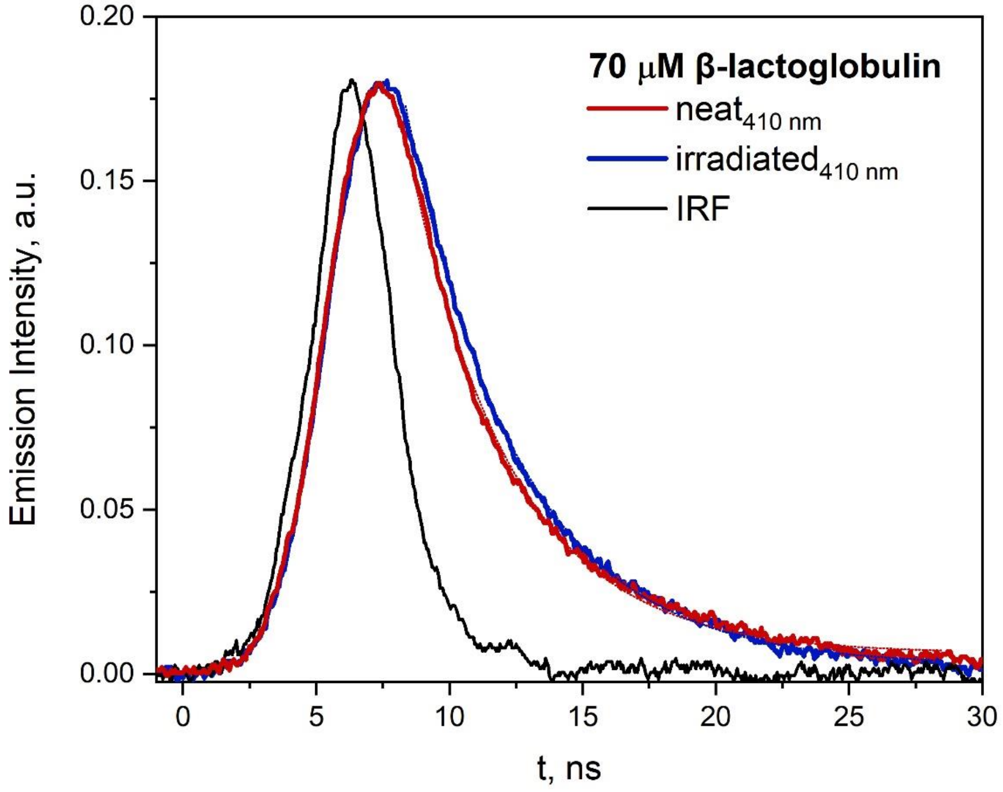

Figure S6. Decays of fluorescence of the neat β-lactoglobulin (70 μM) solution and solution of β-lactoglobulin (70 μM) containing NaN_3 (0.1 M) obtained for irradiation dose 200 Gy (red and blue lines, respectively). Fitting curves (dotted lines, 3.55 ns and 3.76 ns) superimposed on the experimental runs (noisy lines).

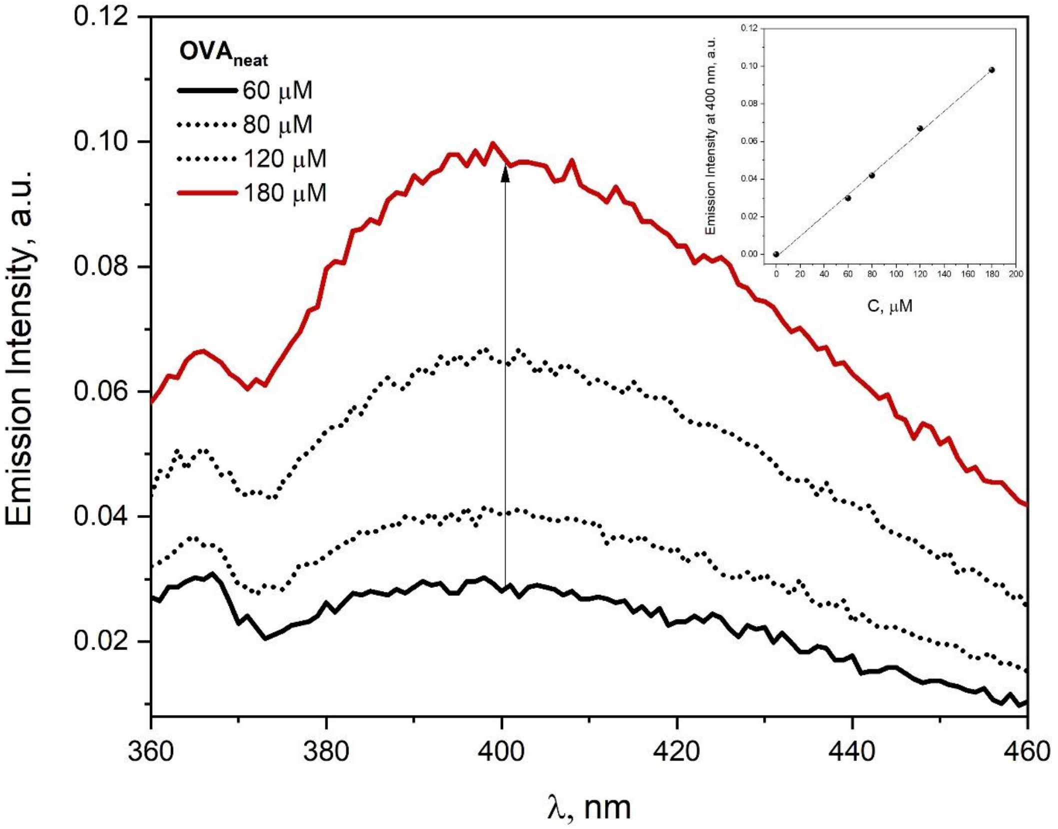

Figure S7. Emission spectra of a neat OVA solutions (60–180 μM). The excitation wavelength was 320 nm. **Insert**: Dependence of I_{max} as a function of OVA concentration. I_{max}—intensity of OVA emission in buffer solution at maximum of emission band, λ_{exc} = 320 nm.

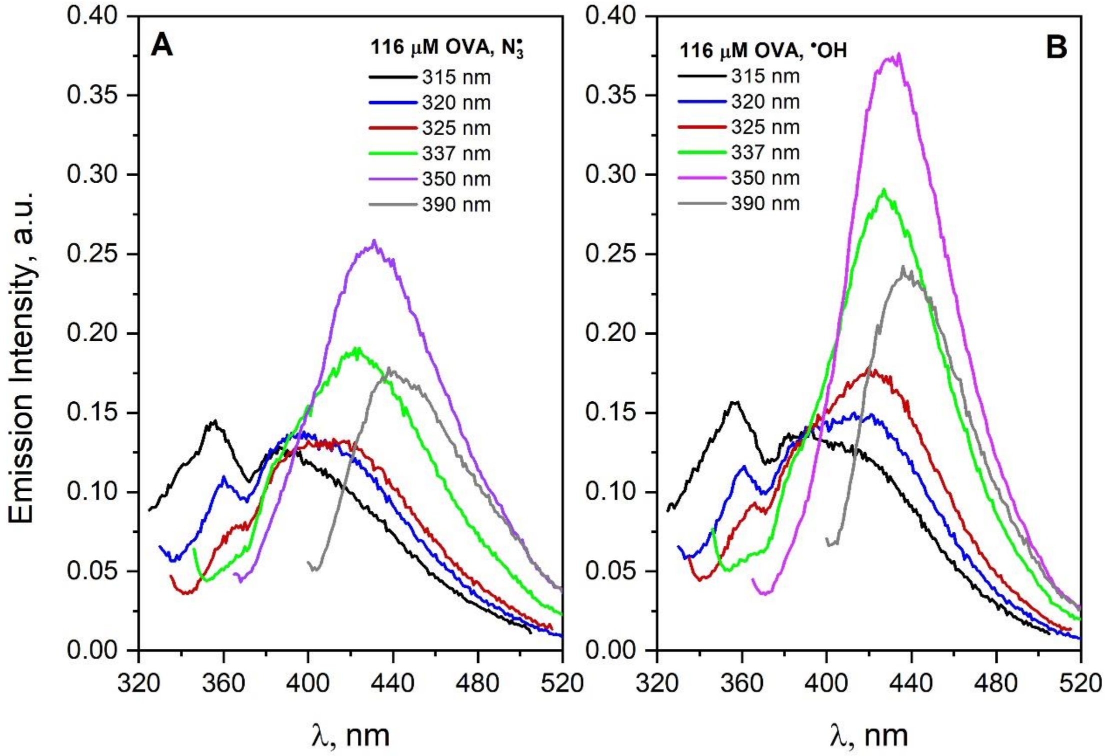

Figure S8 (A). Emission spectra of the N_2O-saturated OVA solution (116 μM) containing NaN_3 (0.1 M), recorded after irradiation with a dose 2200 Gy. The excitation wavelengths are given in the figure. **(B).** Emission spectra of the N_2O-saturated OVA solution (116 μM), recorded after irradiation with a dose 2200 Gy. The excitation wavelengths are given in the figure.

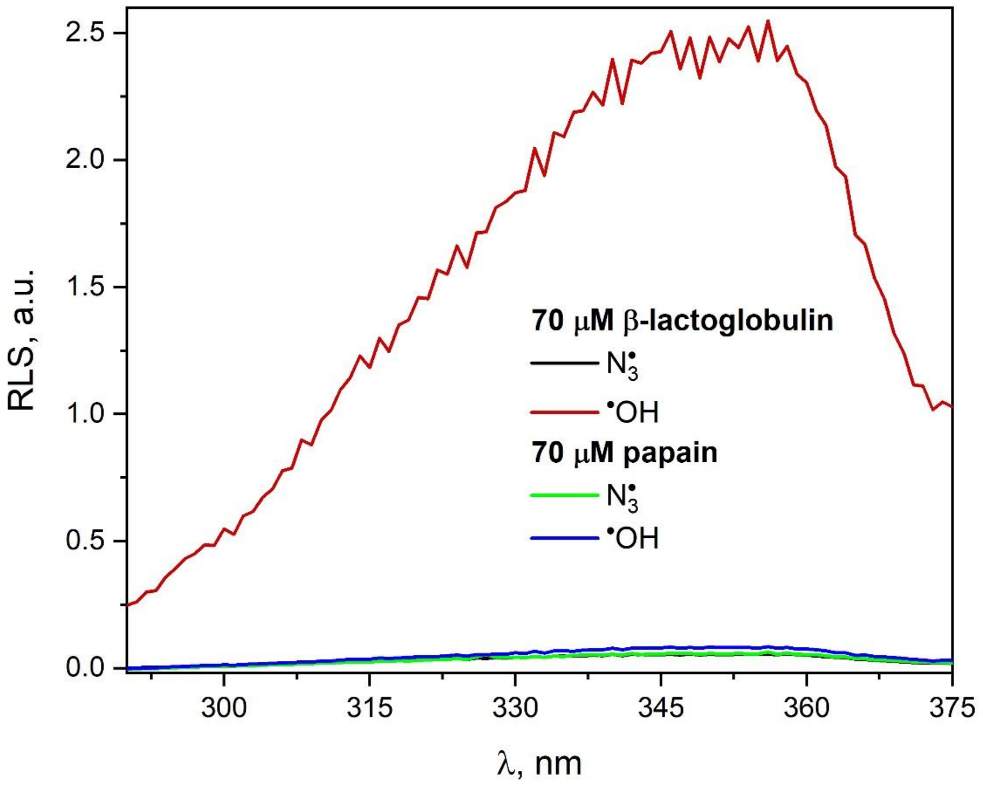

Figure S9. The RLS spectra of N_2O-saturated buffer solution containing 70 μM β-lactoglobulin or 70 μM papain, obtained for an irradiation dose of 5600 Gy. The RLS spectra of N_2O-saturated buffer solution containing 0.1 M NaN_3 and 70 μM β-lactoglobulin or 70 μM papain, obtained for an irradiation dose of 5600 Gy.

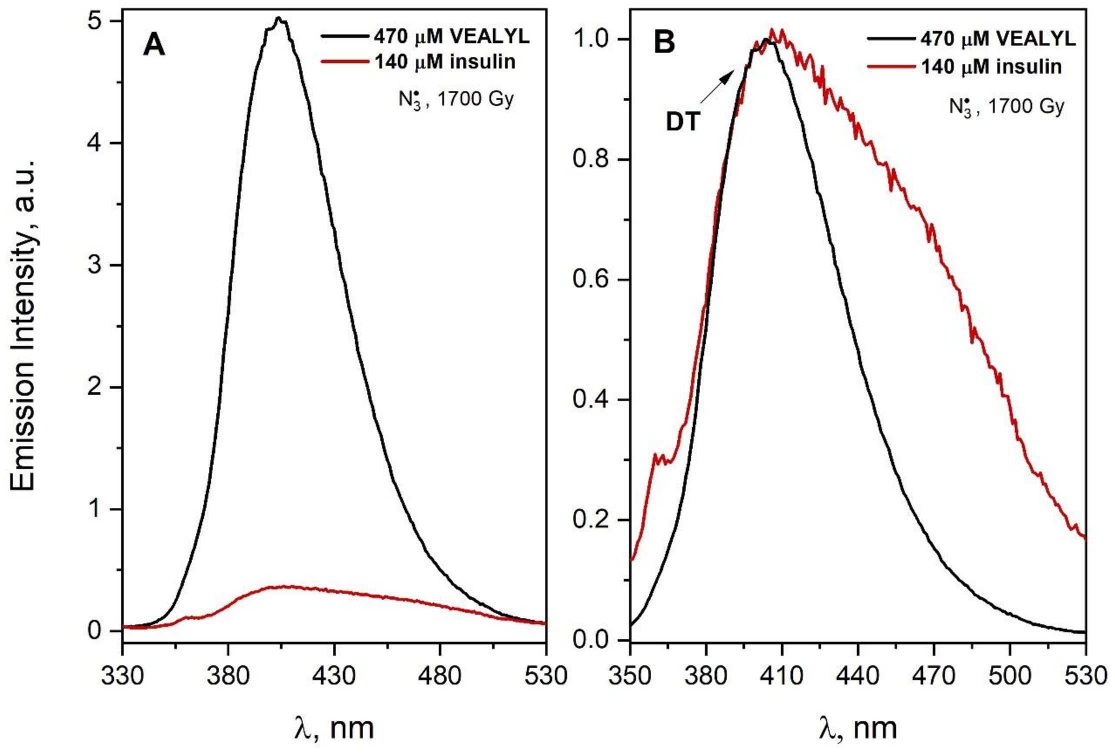

Figure S10 (A). Emission spectra of N_2O-saturated buffer solution of VEALYL (470 μM) or insulin solution (140 μM) containing NaN_3 (0.1 M), recorded after irradiation with dose 1700 Gy. The excitation wavelength was 320 nm. **(B).** Normalized emission spectra of N_2O-saturated buffer solution of VEALYL (470 μM) or insulin solution (140 μM) containing NaN_3 (0.1 M), recorded after irradiation with dose 1700 Gy. The excitation wavelength was 320 nm.

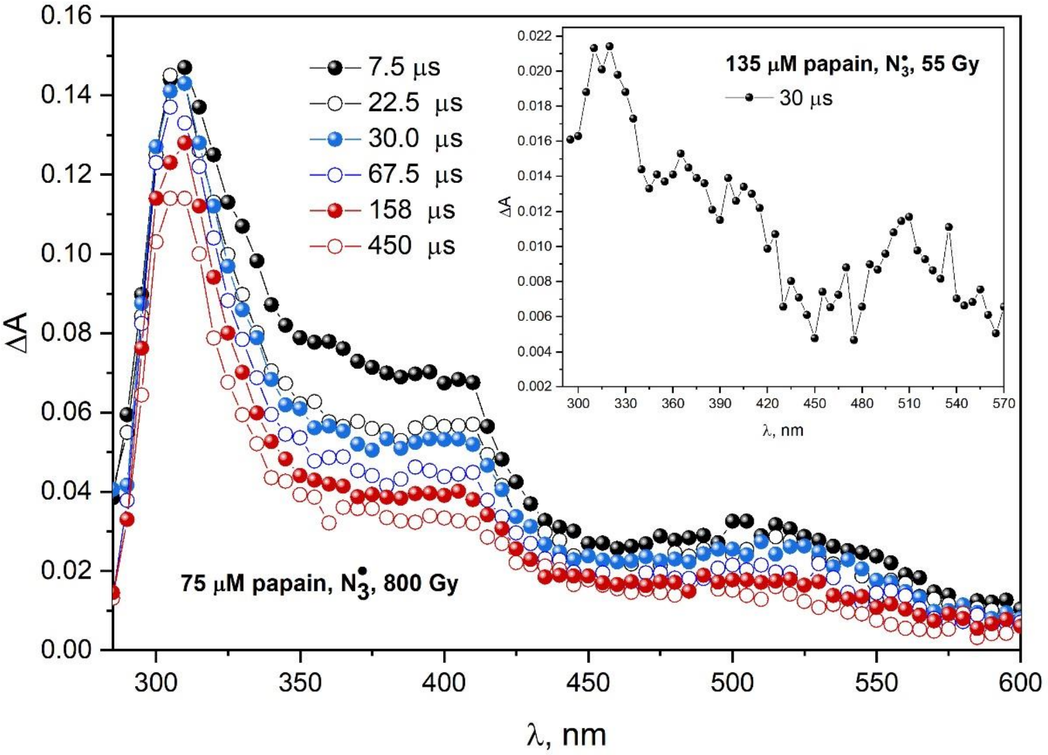

Figure S11. Transient absorption spectra of N_2O-saturated buffer solution containing 75 μM papain and 0.1 M NaN_3, obtained for an irradiation dose of 800 Gy. **Insert**. Transient absorption spectra of N_2O-saturated buffer solution containing 135 μM papain and 0.1 M NaN_3, obtained for an irradiation dose of 55 Gy.

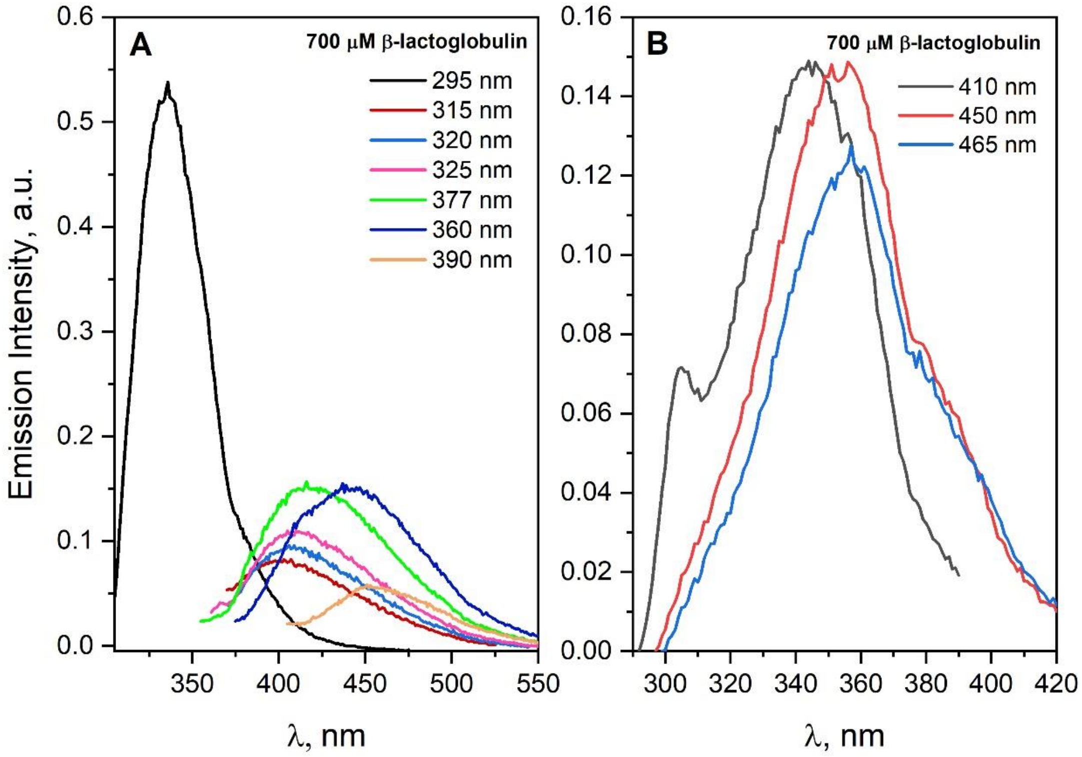

Figure S12 (A). Emission spectra of the neat β-lactoglobulin solution (700 μM). The excitation wavelengths are given in the figure. **(B)** Emission excitation spectra of the neat β-lactoglobulin solution (700 μM). The emission wavelengths are given in the figure.
